# Cost-effectiveness of paclitaxel, doxorubicin, cyclophosphamide and trastuzumab versus docetaxel, cisplatin and trastuzumab in new adjuvant therapy of breast cancer in china

**DOI:** 10.1186/s12962-021-00264-w

**Published:** 2021-02-23

**Authors:** Qiaoping Xu, Li Yuanyuan, Zhu Jiejing, Liu Jian, Li Qingyu, Chen Lingya, Luo Ying, Shi Changchen, Li Yangling, Yan Wei

**Affiliations:** 1 Department of Pharmacy, Hangzhou First People’s Hospital, School of Medicine, Zhejiang University, Hangzhou, 310006 China; 2 Center for Healthcare Security Dig Data and Health Policy Studies, School of Public Health, Zhejiang University School of Medicine, 866 Yuhangtang Road, Hangzhou, 310058 Zhejiang China; 3 Zhejiang Cancer Hospital, Hangzhou, 310022 Zhejiang China; 4 Hangzhou Senile Hospital, Hangzhou, 310022 Zhejiang China

**Keywords:** Breast cancer, Neoadjuvant chemotherapy, Pharmaceutical economics, Markov model, Cost-effectiveness analysis

## Abstract

**Background:**

Breast cancer is the most common cancer among women in China. Amplification of the Human epidermal growth factor receptor type 2 (HER2) gene is present and overexpressed in 18–20% of breast cancers and historically has been associated with inferior disease-related outcomes. There has been increasing interest in de-escalation of therapy for low-risk disease. This study analyzes the cost-effectiveness of Doxorubicin/ Cyclophosphamide/ Paclitaxel/ Trastuzumab (AC-TH) and Docetaxel/Carboplatin/Trastuzumab(TCH) from payer perspective over a 5 year time horizon.

**Methods:**

A half-cycle corrected Markov model was built to simulate the process of breast cancer events and death occurred in both AC-TH and TCH armed patients. Cost data came from studies based on a Chinese hospital. One-way sensitivity analyses as well as second-order Monte Carlo and probabilistic sensitivity analyses were performed.The transition probabilities and utilities were extracted from published literature, and deterministic sensitivity analyses were conducted.

**Results:**

We identified 41 breast cancer patients at Hangzhou First People’s Hospital, among whom 15 (60%) had a partial response for AC-TH treatment and 13 (81.25%) had a partial response for TCH treatment.No cardiac toxicity was observed. Hematologic grade 3 or 4 toxicities were observed in 1 of 28 patients.Nonhematologic grade 3 or 4 toxicities with a reverse pattern were observed in 6 of 29 patients. The mean QALY gain per patient compared with TCH was 0.25 with AC-TH, while the incremental costs were $US13,142. The incremental cost-effectiveness ratio (ICER) of AC-TH versus TCH was $US 52,565 per QALY gained.

**Conclusions:**

This study concluded that TCH neoadjuvant chemotherapy was feasible and active in HER2-overexpressing breast cancer patients in terms of the pathological complete response, complete response, and partial response rates and manageable toxicities.

## Background

Breast cancer is one of the most common malignant tumors in women, and its incidence continues to rise globally. In the worldwide, about 1.2 million women have breast cancer every year, accounting for 18% of all female tumors. The incidence of breast cancer in China also shows an increasing trend year by year [1]. Breast cancer remains the most prevalent invasive cancer among women.China is no exception:

figures showed that the five most commonly diagnosed cancers among women were breast,lung and bronchus,stomach,colorectal,and esophagus cancer.

Trastuzumab, a humanized monoclonal antibody that targets the extracellular domain of the HER-2 protein, was found to improve survival in the metastatic disease setting when used in combination with chemotherapy [2]. Alone and in combination with chemotherapy,trastuzumab has been shown to be safety and active in advanced HER2-positive disease. Then, in a large, randomized study, the addition of trastuzumab to chemotherapy improve the rates of objective response, response duration, and time to disease progression, as well as a 30% improvement in the rate of overall survival among patients with first-line metastatic disease [2]. A significant side effect was cardiac dysfunction, including congestive heart failure,especially when trastuzumab was used in combination with anthracycline-based regimens [2].

At a median follow up of 36 months, the BCIRG 006 trial [3] had recently demonstrated that every 3 weekly Doxorubicin/Cyclophosphamide (AC) followed by the Paclitaxel (T) group had nominally fewer DFS events and OS events than the Docetaxel/Carboplatin/Trastuzumab (TCH) group, but the differences were not statistically significant and the toxicity profiles differed especially regarding cardiac events. In the BCIRG006 trial (trastuzumab plus chemotherapy in Early Breast Cancer [EBC]), the incidence of congestive heart failure was 2.0 and 0.4% in the anthracycline and nonanthracycline arms, respectively [3]. It was also anticipated that TCH might have less cardiac toxicity than anthracycline-based regimens [2].

Randomized Phase III Study of TCH in patients with advanced disease showed that this regimen resulted in the longest period of objective response rate (ORR) and progression free survival (PFS) in women with HER-2-overexpressing metastatic breast cancer(MBC), with rare cardiac dysfunction [4].

In prospective studies, Russell et al. [5] summarized that the incidence of symptomatic heart failure events was 2.0% in patients treated with AC-TH,and the majority of these patients recovered with appropriate treatment. In the same year, another paper also reported that the incidence of heart failure (CHF) in patients treated with AC-TH [6] was 2% to 4%. The risk benefit ratio favored the nonanthracycline TCH regimen over AC-T plus trastuzumab, given its similar efficacy, fewer acute toxic effects, and lower risks of cardiotoxicity, and the incidence of the heart failure (CHF) was 0.4% [7, 8]. Therefore, docetaxel, carboplatin, and trastuzumab (TCaH) is an option for HER2-positive patients who can not use anthracycline. However, carboplatin toxicity could be a concern in certain patients. The primary determinant of carboplatin clearance was the glomerular filtration rate (GFR), which was often decreased in elderly patients and may increase the risk of toxicity [9].

To determine the worth and safety of omitting an anthracycline, a randomized trial of dose-dense AC followed by TH compared with DCT (TCH) could be considered. Therefore, we reviewed our experience concerning the safety and tolerability of standard trastuzumab-containing chemotherapy regimens (AC-TH and TCH) in early-stage, resectable, HER2-positive breast cancer patients. The aim of this paper was to evaluate the cost effectiveness of adjuvant AC-TH compared with TCH in the hospital setting for women with early (regional) breast cancer to help guide funding decisions for incurring breast cancer.

## Methods

### Statement

According to the ministry of health "measures on ethical review of biomedical research involving human beings (trial 2007)"、WMA《Declaration of Helsinki》and the ethical principles of CIOMS《the international moral guide to human biological research》,Subject to review by the ethics committee, Agree to carry out this study according to the research scheme under review.

All methods were carried out in accordance with relevant guidelines and regulations. All experimental protocols were approved by Ethics committee of hangzhou first people's hospital. Informed consent was obtained from all subjects. The protocol of the trial was registered prior to initiation. (The trial registration number: [2014] Scientific Research Medical Ethics No. (028) -01).

### Design and structure of the model

**Fig 1.**
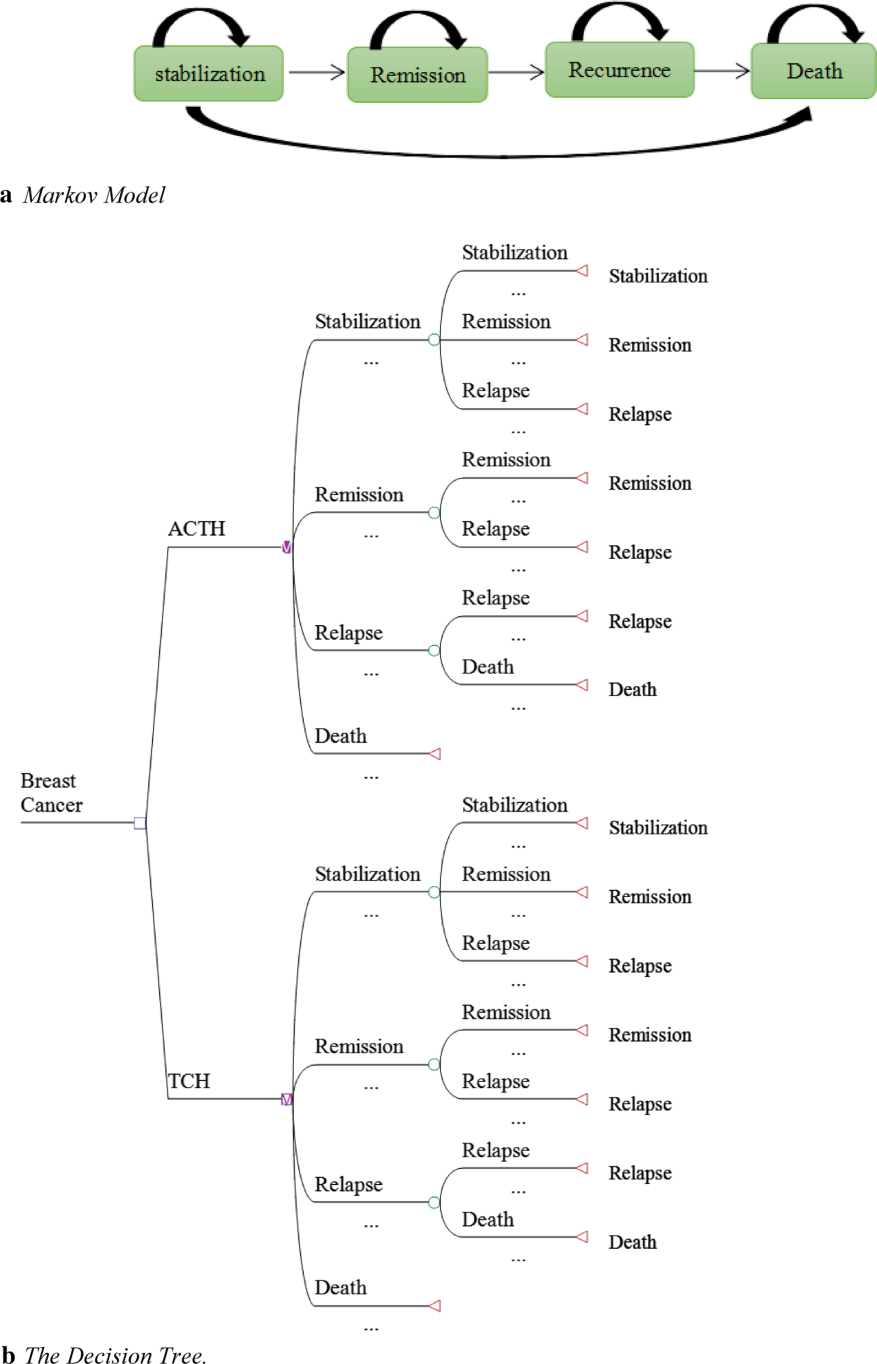
Schematic of the Decision Tree and Markov Model. *ACTH* Doxorubicin/Cyclophosphamide/Paclitaxel/Trastuzumab, *TCH* Docetaxel/Carboplatin/Trastuzumab)

We constructed a Markov model using TreeAge Pro Suite 2011 (TreeAge Software Inc., Williamstown, MA, USA) to analyze the cost-effectiveness of a novel adjuvant AC-TH to the TCH treatment for patients with HER-2-positive breast cancer. In the model, presented in Fig. 1, the patients move between the following four health states: remission, stabilization, relapse and death. Movements between health states were based on transition probabilities that were calculated from clinical trials with a one-year cycle length and adjusted to half cycle in each health state process. The time horizon was five years based on the clinical literature.I state that the probabilities came from literature.

Patients accumulated both the costs and quality-adjusted life-years (QALYs) for each period spent in a certain health state. We discounted both the costs and QALYs at a 5% rate. Since the time value of money is different, discount rate has a certain influence on the calculation of cost. In the baseline analysis, 5% discount rate is used to calculate the cost, and in the sensitivity analysis, 0%-5% discount rate is used to calculate the incremental cost utility ratio, so as to investigate the stability of the results.The results were presented in incremental cost-effectiveness ratios (ICERs) in terms of the cost per QALY gained. To perform probabilistic sensitivity analysis (PSA), we simulated the model using second-order Monte Carlo analysis (1000 simulations) and constructed confidence intervals with bootstrap replications (1000 replications).

### Patients and treatment plans

We identified 41 patients treated with neoadjuvant trastuzumab using our institutional database from January 2014 to November 2016. All the patients were confirmed to have HER2-positive breast cancer by either immunohistochemistry or fluorescence in situ hybridization. Chemotherapy regimens were selected according to physician preference. Twenty-five patients received AC-TH, and sixteen patients received TCH.

Patients in the AC-TH group received doxorubicin (60 mg/m^2^) and cyclophosphamide(600 mg/m^2^) every 2 or 3 weeks for four cycles, followed by weekly paclitaxel (80 mg/m^2^) for 12 weeks. Trastuzumab was administered with the first dose of paclitaxel at a dose of 4 mg/kg body weight, followed by 2 mg/kg body weight weekly during chemotherapy. In the TCH regimen, patients received 75 mg/m^2^ of docetaxel and 6 mg per ml/min of carboplatin (area under the curve) once every three weeks for six cycles. Trastuzumab was given concurrently with this treatment and consisted of a 4 mg/kg loading dose followed by 2 mg/kg weekly for 12 weeks. Thereafter, the dosage became 6 mg/kg once every three weeks until a one-year course was completed.

Patients with a high risk of febrile neutropenia treated with standard AC-TH (every 3 weeks)received granulocyte-colony stimulating factor (G-CSF) [23]. Patients who developed asymptomatic cardiotoxicity(decrease in left ventricle ejection fraction) were treated with a course of angiotensin-converting enzyme (ACE) inhibitors and another echo. Patients who experienced symptomatic cardiotoxicity (congestive heart failure) were treated with ACE inhibitors, beta blockers and another echo [24] such as dexrazoxane.

### Model inputs for transition probabilities

Transition probabilities, sourced from clinical trials, are presented in Table 1. All the hazard ratios and survival rates were converted into transition probabilities for one-year time periods.

**Table 1:** Key parameters for the model of adjuvant treatment of regional breast cancer with ACTH and TCH

Variable	Formula	Best estimate	SA range	Distribution	Source
Probability					
–ACTH RR		0.837			[14–18]
OS TTP		44.3 7.2			
DOR		5.6			
Stable → Stable (ACss)	1-ACsp-ACsr	0.401	0.3208–0.4812	Beta	
Stable → Remission (ACsr)	1-exp(−RR/3)	0.243	0.1944–0.2916	Beta	
Stable → Relapse(ACsp)	ACrp*4	0.356	0.2848–0.4272	Beta	
Remission → Remission (ACrr)	1-ACrp	0.911	0.7288–1.0000	Beta	
Remission → Relapse (ACrp)	exp(− 0.75*In (2)/(5.6))	0.089	0.0712–0.1068	Beta	
Relapse → Relapse (ACpp)	1-ACpd	0.986	0..7888–1.0000	Beta	
Relapse → Death (ACpd)	exp(− 0.75*In (2)/(44.3–7.2))	0.014	0.0112–0.0168	Beta	
−TCH RR		0.704			[19–23]
OS TTP		35 10.35			
DOR		4			
Stable → Stable (TCss)	1-TCsp-TCsr	0.311	0.2488–0.3732	Beta	
Stable → Remission(TCsr)	1-exp(− RR/3)	0.209	0.1672–0.2508	Beta	
Stable → Relapse(TCsp)	TCrp*4	0.48	0.384–0.576	Beta	
Remission → Remission (TCrr)	1-TCrp	0.88	0.704–1.0000	Beta	
Remission → Relapse (TCrp)	exp(− 0.75*In (2)/4.2))	0.12	0.096–0.144	Beta	
Relapse → Relapse(TCpp)	1-TCpd	0.979	0.7832–1.0000	Beta	
Relapse → Death(TCpd)	exp(− 0.75*In (2)/(35–10.35))	0.021	0.0168–0.0252	Beta	
Discount rate for costs and QALYs		5% per year			[24]
Health state utilities					[13]
No recurrence (chemotherapeutic period)		0.74	0.592–0.888	Beta	
No recurrence (after chemotherapy)		0.94	0.752–1.000	Beta	
local recurrence (in the first year)		0.74	0.592–0.888	Beta	
remission		0.85	0.68–1.000	Beta	
Relapse		0.5	0.4–0.6	Beta	

*OS* overall survival, *DOR* duration of response, *TTP* time to progression

We calibrated the transition probabilities from our actual overall survival (OS) data and median time to progression using the equation developed by the log-logistic models [10–12] and inputed into the Markov model. The modeled graphical OS data were extracted using a validated graphical digitizer (Web Plot Digitizer version 3.4; Ankit Rohatgi, Austin, TX, USA). The difference between actual OS data and model data derived from our Markov states was compared using chi-squared test. The results represented the internal validation of our model (See Table 2). Health state utilities referred to those used in previously published cost-effectiveness analyses of trastuzumab [13]. We assumed these hazard ratios for disease-free survival corresponded to a five-year period, similar to the BCIRG 006 data for trastuzumab.

**Table 2:** Cost and effect of ACTH and TCH within five years

Item	Status	ACTH	TCH	Deviation
Effect	Disease Free Survival/%	38%	46%	− 8
	Death/%	2.6%	1.4%	1.2
	QALY/Year	3.4	3.65	− 0.25
Intervention costs^a^($)		3112	1352	
Health system costs($)		56,425	45,043	
Cost($)		59,537	46,395	13,142
ICER				− 52,568

^a^Other costs included were those of the prevention and treatment of febrile neutropenia and peripheral neuropathy

### Model inputs for effectiveness

The utilities that used in the model are presented in Table 1. Values for the utility associated with health states were taken from a study by Ward et al. [13]. These utilities were chosen because of the similarities in setting.

### Model inputs for costs

All the drug costs came from the Hangzhou First People’s Hospital, China. The costs of the treatments were calculated considering the patient body weight and body surface area. We assigned costs attributable to chemotherapy to the first cycle in the Markov model.

### Simulations and uncertainty

By calculating a weighted average using the heterogeneity distribution, incremental QALYs and costs were obtained for all expected breast cancer patients diagnosed in 2015. The analyses were performed using TreeAge Pro 2011.

Monte Carlo simulation was used to address parameter uncertainty, with 1000 draws from input parameters based on the following distributions: log-Normal distribution for the HR for death; beta distributions for the proportions experiencing a toxicity and disability weights; gamma distributions for costs (see Table 2). We reran the models (expected values only; no uncertainty about input parameters) for a range of scenarios to assess the impact of the following structural assumptions:

- discount rate 0 or 5% per annum.
- exclusion of prevalent life-years with QALYs.
- exclusion of unrelated health system costs.

Additionally, we relied on these expected value-only analyses for incremental cost-effectiveness ratios (ICERs). Similar to parameter uncertainty, many simulations resulted in negative QALYs and sometimes also negative costs, making the mean and median ICERs difficult to interpret.

We also performed a range of one-way sensitivity analyses, using the 2.5th and 97.5th percentile values of the input parameters to assess which ones contributed the most to uncertainty in the model QALYs and incremental cost outputs, but not the ICERs for the reason given above.

Finally, we also performed net monetary benefit (NMB) analysis and produced cost-effectiveness acceptability curves using these NMB values rather than ICERs because of the greater validity of NMBs in this instance given the occurrence of negative ICERs. A cost-effectiveness threshold of $US 34,240 per QALY was set for NMB analysis. This threshold equated to the China gross domestic product (GDP) per capita in 2015.

### Sensitivity analysis

To perform probabilistic sensitivity analysis, we assigned distributions to the model parameters to represent the uncertainty associated with point estimates. We applied Monte Carlo simulation to generate 1000 runs and identified the 25th and 97.5th ranks as the corresponding 95% confidence intervals (CIs).A beta-distribution was used to represent the uncertainty in utility,probability and proportions because these are binomial parameters that are constricted in the interval from zero to one. A gamma-distribution was used for cost data.Based on the results of PSA, a cost-effectiveness acceptability curve was plotted to show the proportion of cost-effective simulations at different levels of willingness to pay per QALY gained.

Moreover, We also performed one-way sensitivity analyses. We varied the age at diagnosis and evaluated the impact of changes to assumptions regarding treatment decisions and the target population. We also extended the time period during which patients were at risk for recurrence, varied the discount rate, applied utility weights from other sources, applied wages of government workers to value the patient time, doubled the cost assigned for chemotherapy, and applied higher costs for distant recurrence.

## Results

### Base-case treatment analyses

Our model calculated the lifetime cumulative costs, quality adjusted life years.

(QALYs), incremental QALYs, incremental costs, incremental cost-effectiveness ratios(ICERs), and incidences of death for the five treatment strategies (Table 2).

**Fig. 2:**
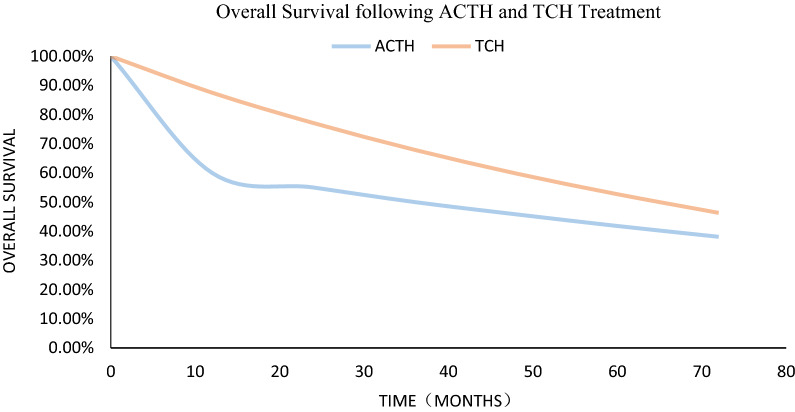
Overall survival. The curves indicate the model outputs. The diamond and triangle markers indicate the 5-y OS of ACTH and TCH treatment

The Markov model calculated that the four distribution proportions of the ACTH program were stabilization (1.1%), remission (38.2%), relapse (58.2%) and death (2.6%). Additionally, the four distribution proportions of the TCH program were stabilization (0.2%), remission (46.3%), relapse (52.1%) and death (1.4%). The 5-y OS of TCH treatment was more than that of ACTH treatment (see Fig. 2).

Under our baseline assumptions, the model results showed that TCH was the dominant strategy: it was both less costly and offered more QALYs than the ACTH regimen. On average, TCH cost $13,142 < ACTH. The effectiveness analysis showed that TCH was slightly preferred over the ACTH strategy when measured in QALYs (a difference of 0.25 QALYs). In the treatment of ACTH, PR of the breast cancer was achieved in 15 patients (60%), stable disease was achieved in 3 patients (12%), and seven patients (28%) progressed. However, in the treatment of TCH, CR of the breast cancer was achieved in 1 patient (6.25%), PR of the breast cancer was achieved in 13 patients (81.25%), stable disease was achieved in 1 patient (6.25%), and 1 patient (6.25%) progressed (See Table 2).

The QALYs gain was greater with TCH than with ACTH, with an estimated incremental QALY gain of 0.25 for ACTH versus TCH (Table 2). Additionally, the treatment of ACTH had greater costs. However, the extreme of the uncertainty intervals (UIs, i.e., the negative 2.5th percentiles) included QALY losses (Table 2). The costs were also higher with ACTH than with TCH. We used expected value analysis, where the incremental costs and QALYs were the same or close to the averages from the Monte Carlo simulations.

**Fig 3.**
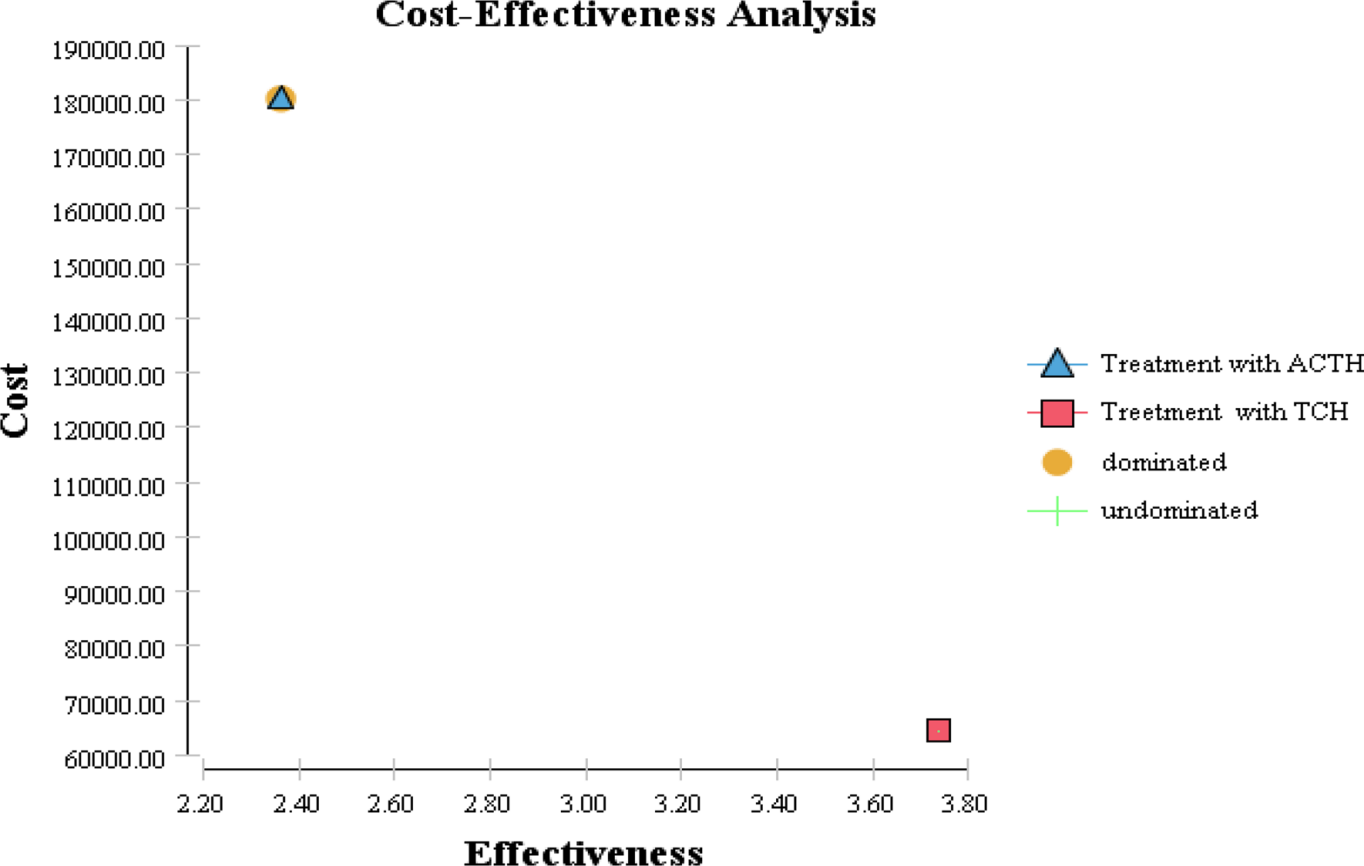
Results of cost-effectiveness analyses for the Breast cancer patients. The vertical axes represent the lifetime cumulative cost and the horizontal axes represent the quality-adjusted life years (QALYs) gained. The AC-TH strategy is the dominant strategy because it is the most effective and the least expensive of the two alternative strategies

The economic outcomes of alternative strategies are presented in Fig. 3. The AC-TH strategy is the dominant strategy because it is the most effective and the least expensive of the two alternative strategies. Applying incremental analysis principles, the ICER for ACTH versus TCH was $US52,568 per QALY gained (See Table 2).

### General safety

The most common AEs (any grade) during the neoadjuvant period were anemia, infection, hepatotoxicity, renal toxicity, cardiac toxicity and neutropenia (Table 3). The most common grade 3–4 AEs were infection, hepatotoxicity and neutropenia (Table 3), consistent with the higher proportion of patients who received optional prophylactic bone marrow support in ACTH. Neutropenia recorded as “neutrophil count decreased” was reported in 7 patients (28%) in the ACTH group and 1 patient (6%) in the TCH group. No grade 5 AEs were reported. The most common serious AEs were hepatotoxicity and infection. Ten patients (40%) in the ACTH group experienced grade 3–4 hepatotoxicity, while four patients (25%) in the TCH group experienced grade 3–4 hepatotoxicity. In our study, the incidence of symptomatic cardiotoxicity with ACTH treatment was 32%, which was greater than that with TCH treatment.

**Table 3:** Adverse events (G1-2 and G3-4) for ACTH vs TCH

	ACTH Adverse events		TCH Adverse events	
	G1-2	G3-4	G1-2	G3-4
Anemia	5 (20%)	4(16%)	4(25%)	7(44%)
Infection	3 (12%)	18(72%)	0	5(31.25%)
Neutropenia	5 (20%)	7(28%)	2(12.5%)	1(6%)
Hepatotoxicity	2 (8%)	10(40%)	1(6%)	4(25%)
Renal toxicity	2 (8%)	0	2(12.5%)	0
Cardiac toxicity				
Symptomatic dysfunction	8 (32%)	0	3(18.75%)	0
Cardiac failure	0	0	0	0

### Sensitivity analyses

#### Single-factor sensitivity analysis

**Fig. 4:**
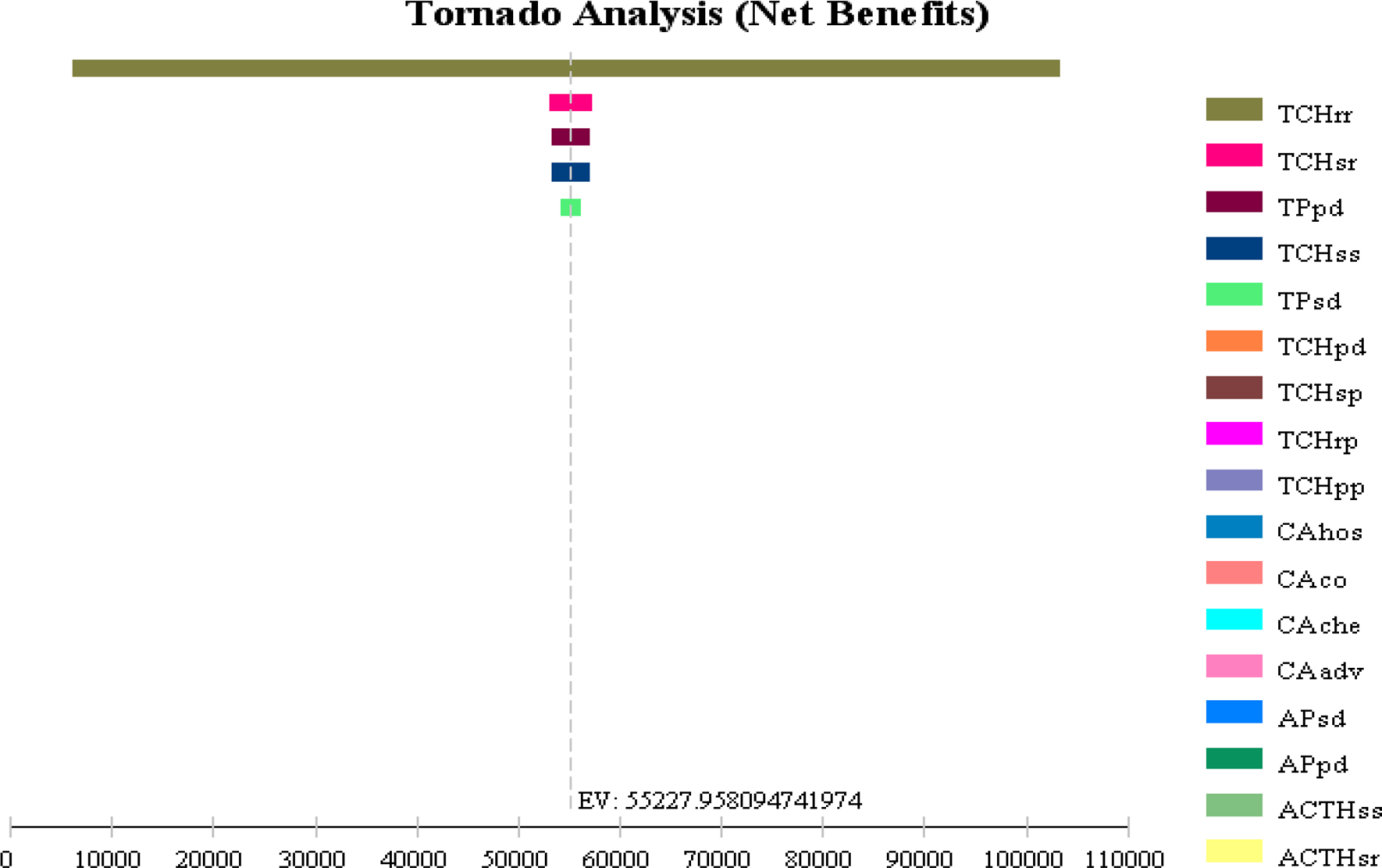
Tornado diagram representing the cost per QALY gained in one-way sensitivity analysis for AC-TH strategy versus TCH strategy.The width of the bars represents the range of the results when the variables were changed. The width of the bars represents the range of results when the variables are changed.The vertical dotted line represents the base-case results

Because of the method of calculating the transfer probability in this study was based on the literature data, the value of the corresponding transfer probability calculated from the maximum and minimum value in the literature data was used as the range of sensitivity analysis. Single-factor sensitivity analysis was conducted using the sensitivity analysis method provided by TreeAge software. For both chemotherapy regimens, the transfer probability of remission to progression has the greatest impact on the model results. Using the tornado diagram function of TreeAge software, the tornado diagram of the transition probability is drawn, as shown in Fig. 4. The figure shows that the transition probability of the slow transfer to the progress state is an important variable influencing the conclusion of the model; thus, the variation of each transition probability within its sensitivity range would affect the change in the conclusion.

Because the drug prices in the study was from a hospital’s data and the incidence of adverse reactions, adverse reactions costs were derived from real data, health utility data was from the literature.So all these parameters input in the Markov model conducted by single-factor sensitivity analysis using the sensitivity analysis method. To test the influence of these variables in the results of cost-effectiveness analysis.The tornado diagram of the transition probability is drawn, as shown in Fig. 4. The outcome of the one-way sensitivity analysis is reported as a “tornado diagram” (Fig. 4), and only the parameters that accounted for 99% of the cumulative risk related to the ICER are displayed.

The one-way sensitivity analyses revealed that some model parameters have a substantial impact on the results.The 5 most influential parameters are: the transition probability of TCH scheme from remission to remission (TCHrr), and the transition probability of TCH scheme from stabilization to remission (TCHsr), the transition probability (TPpd) of TCH scheme, the probability of transition from stable to stable of TCH scheme(TCHss), the transition probability of TCH scheme (TPsd).

Nevertheless,other parameters,including drug costs and other disease utilities,had little impact on the robustness of the model. When the threshold was set at $20,000 per QALY, the above parameters have a certain influence on the ICER results in the sensitivity analysis range.

Because the health utility value of this study is only from a foreign literature, this parameter is unstable,but after sensitivity analysis of various parameters, it is concluded that the parameters that have a greater impact on the research results are mainly the TCH group probability of transition from remission to remission.

#### Results of probability sensitivity analysis

**Fig. 5:**
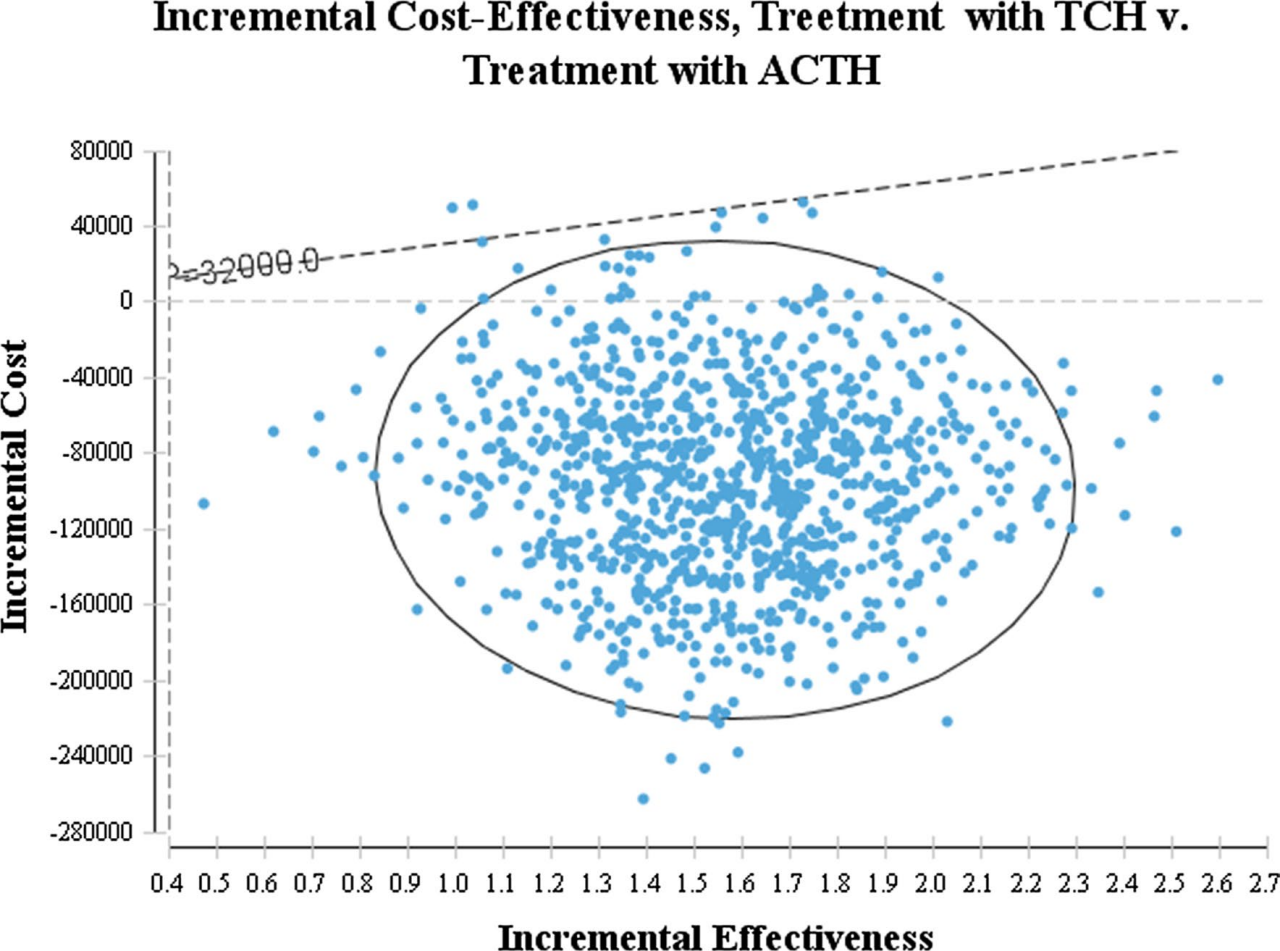
Probabilistic results of the incremental cost-effectiveness differences between treatment with ACTH and TCH for a cohort of 1000 Breast cancer patients.The vertical axes represent the incremental costs.The horizontal axes represent the incremental quality-adjusted life years (QALYs) gained.The data points below the willingness-to-pay (ceiling ratio) threshold represented by the oblique line reflect simulations in which the cost per QALY gained with TCH treatment was below the Chinese cost-effectiveness (C/E) threshold of $20,000. For example, the TCH strategy would be cost-effective when compared with alternative strategies if a patient treated with TCH gained 1 incremental QALY and the incremental cost was lower than $20,000

The probability sensitivity analysis uses the Monte Carl simulation method. Each simulation generates a Markov cohort of 10,000 people. The total number of Markov cohorts is 1000. After calculation,the two ICERs of neoadjuvant chemotherapy for breast cancer are compared. Furthermore,a probability sensitivity analysis can be performed. According to the Monte Carlo simulation results,the scatter plots of the TCH scheme and the ACTH scheme are compared. The horizontal and vertical axes represent the incremental effect and incremental cost of the TCH scheme compared with the ACTH scheme. Each scatter in the figure represents the TCH scheme. The incremental cost-effectiveness ratio (ICER) of breast cancer with ACTH regimen is shown in the figure. Most of the scattered points in the figure are distributed in the first and fourth quadrants. The ICER ratio is generally negative,which shows that the TCH scheme has absolute advantages (see Fig. 5).

**Fig. 6:**
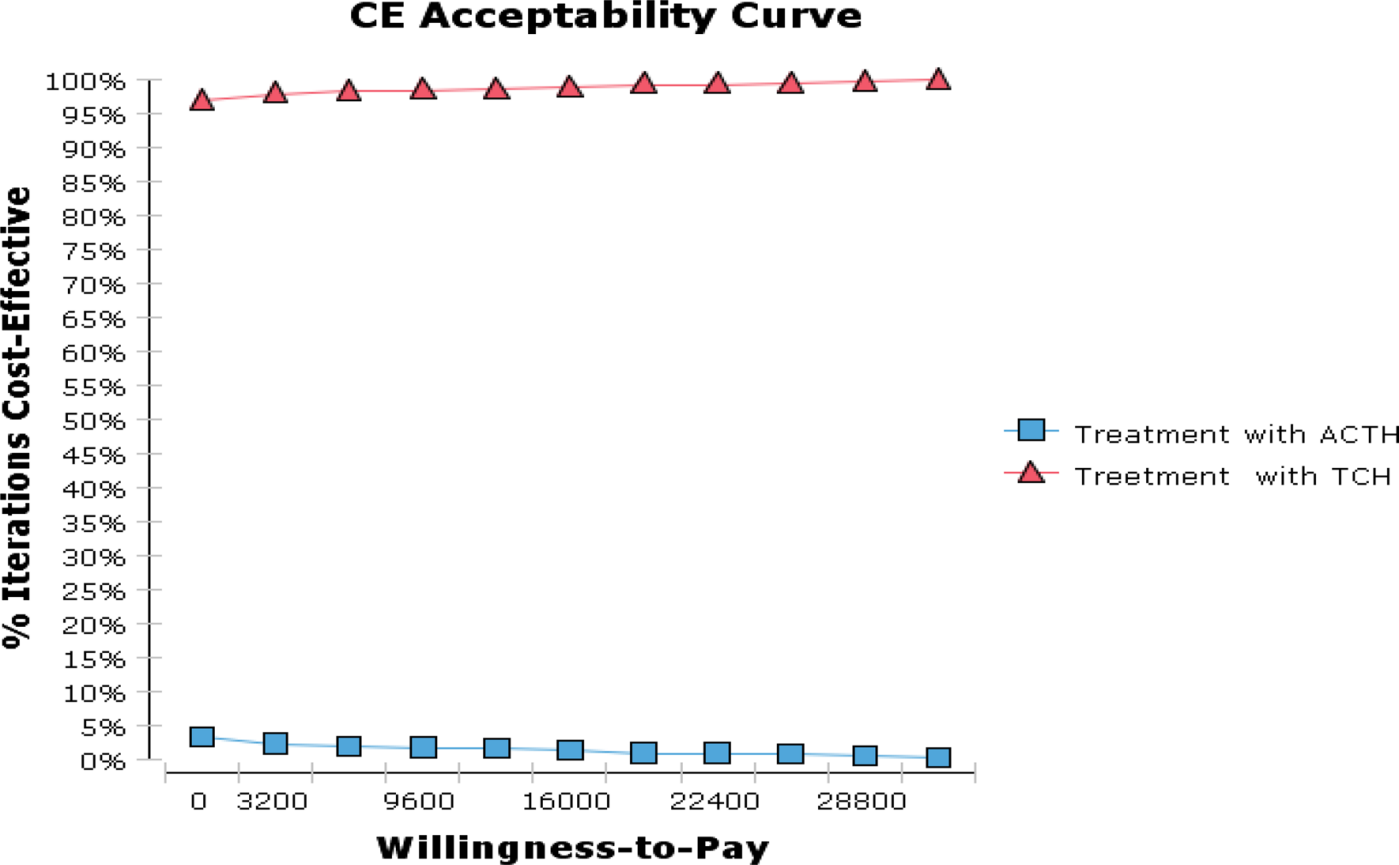
Cost-effectiveness acceptability curves showing the probabilities of net benefits achieved by each strategy for different willingness-to-pay thresholds (the maximum amount a person would be willing to pay for a good) in ACTH (a) and TCH(b) cohorts. The vertical axes represent the probability of cost-effectiveness. The horizontal axes represent willingness-to-pay thresholds to gain one additional quality-adjusted life year (QALY). For example, if the threshold was set at $20,000 per QALY, the probability of cost-effectiveness of TCH was highest

For a cohort of 1000 breast cancer patients, the cost-effectiveness acceptable curve can be derived from the mutual comparison of the two schemes. It can be seen from Fig. 6 that within the WTP range, the probability of the ACTH scheme has a cost effect which is close to 0, and the TCH scheme acceptance curve is maintained at a high level, which indicates that the TCH scheme has the highest acceptable probability and is the preferred solution.

## Discussion

The incidence of cardiotoxicity was increased when trastuzumab was used in combination with anthracyclines. The BCIRG-006 trial compared three chemotherapy regimens: doxorubicin, cyclophosphamide and docetaxel (AC-T), ACT plus trastuzumab (ACT-H), and docetaxel, carboplatin plus trastuzumab (TCH) for the treatment of HER-2-positive breast cancer [8]. In this trial, both trastuzumab containing regimens (ACT-H and TCH) were superior to ACT and similar to each other in terms of cancer efficacy. Importantly, TCH was associated with significantly less asymptomatic cardiotoxicity (> 10% decline in EF 9.4 vs. 18.6%; *p* < 0.001) and a lower incidence of symptomatic heart failure (0.4 vs. 2%; *p* < 0.001) than ACTH [8]. In our study, the incidence of symptomatic cardiotoxicity of ACTH treatment was 32%, which was more than that of TCH treatment. Additionally, in our study, the patients who had undergone ACTH treatment were also administered dexrazoxane, which is an effective iron chelator that reduces oxygen free radical production when administered with anthracyclines [25]. Swain and colleagues evaluated the data from three prospective studies to determine both the incidence of doxorubicin-related congestive heart failure CHF and the accumulated dose of doxorubicin at which CHF occurred [26]. The patients who received dexrazoxane had a significantly decreased incidence of cardiac events (defined as a decline from baseline LVEF ≥ 20%, decline in LVEF ≥ 10% from baseline and < lower limit of normal, or symptomatic CHF) compared with placebo [26]. Furthermore, they showed that dexrazoxane was cardioprotective even when it was given after patients had already received 300 mg/m^2^ of anthracyclines [27]. In our study, we found that with both ACTH and TCH treatment, none of the patients developed CHF. The cause may be related to the drug conferring a protective effect on the patients’ hearts. However, the FDA currently limits the use of dexrazoxane in women with metastatic breast cancer who need > 300 mg/m^2^ of anthracyclines. Due to concerns regarding decreased tumor response rates, increased myelosuppression and an increased incidence of the development of delayed hematologic malignancies, the routine use of dexrazoxane was not recommended in patients receiving anthraczycline therapy. We had consulted many references concerning dexrazoxane. A subsequent Cochrane meta-analysis had shown no difference in the oncologic response rates or incidence of secondary malignancies between patients receiving chemotherapy with or without dexrazoxane [28]. In this study, 28% of patients treated with the AC-TH regimen had febrile neutropenia, while the percent of febrile neutropenia was 6% in the TCH groups.

Re-evaluation of the cost effectiveness of the ACTH in the adjuvant treatment of breast cancer was timely given the emergence of TCH as a more effective option than the previous standard and significant changes in the relative costs of ACTH. For the first time, to our knowledge, the current economic analysis directly compared the cost effectiveness of ACTH and TCH in the adjuvant treatment of early breast cancer.

The results showed that TCH was likely to be a cost-effective option compared with ACTH, with ICERs less than $US 52,568 per QALY gained. While there was no set cost-effectiveness threshold in China, the ICERs were below the threshold based on the GDP per capita (approximately $US 34,240 in 2015). In order to reduce the risk of death and suffering from disease, $US52,568/QALY should be acceptable to patients in our country.Thus, based on this model, TCH would be the preferred option if the health system was willing to pay the additional cost to benefit from the additional health gain. Indeed, our analysis showed that, at WTP thresholds above $US 20,000 per QALY gained, TCH would have a greater probability of producing a more favorable NMB than ACTH. However, if the WTP threshold was less than this, ACTH would be preferred.

However, to our knowledge, no other cost-effectiveness analysis has directly compared different trastuzumab regimens, and the finding that TCH was cost effective compared with ACTH was not surprising. Jitawatanarat et al. [16] evaluated the tolerability and cardiac safety of docetaxel, cyclophosphamide, and trastuzumab (TCyH) for the treatment of early-stage human epidermal growth factor receptor-2 (HER2)-positive breast cancer and compared the standard trastuzumab-based chemotherapy regimens doxorubicin with cyclophosphamide followed by paclitaxel and trastuzumab (ACTH) and docetaxel, carboplatin, and trastuzumab (TCaH). TCyH was well tolerated and should be investigated as an alternative adjuvant chemotherapy option for patients who are not candidates for standard trastuzumab-containing regimens when both were evaluated against non-taxane regimens [16].

Untch et al. [15] evaluated the efficacy and safety of epirubicin and cyclophosphamide followed by paclitaxel and trastuzumab as neoadjuvant treatment in patients with human epidermal growth factor receptor 2(HER2)-overexpressing breast cancer. Thirty-nine percent of the 217 enrolled patients achieved a pCR. The three-year disease-free survival (DFS) was 88% in patients with pCR compared with 73% in patients without pCR (*P* = *0*.01). The three-year overall survival (OS) was 96% in patients with pCR compared with 86% in patients without pCR (*P* = 0.025). Forty-eight recurrences were reported during a median follow up of 41 months corresponding to an estimated 3-year disease-free survival rate of 77.9%.Twenty-three deaths were reported during post treatment observation, corresponding to an estimated 3-year OS rate of 89.4% [15]. Based on the results of the joint analysis of B-31 and NCCTG N9831, this benefit was considerable. At 4 years, the estimated absolute improvement in disease-free survival is 18% (85−67%; 95% CI, 13–24%), and for overall survival, the benefit was 4.8% (91.4−86.6%; 95% CI, 0.6–9.0%) [29].

Kolberg et al. [21] collected data from adjuvant trials and had shown that the combination of docetaxel, carboplatin and weekly trastuzumab (TCH) was well tolerated and as effective as anthracycline-containing regimens. After a median follow up of 48.5 months, the disease-free survival (DFS) was 84.6%, the distant disease-free survival (DDFS) was 87.2%, and the overall survival (OS) was 91%. Bayraktar et al. [19] evaluated the pathologic complete response (pCR) rates and relapse-free survival (RFS) and overall survival (OS) of patients receiving neoadjuvant systemic therapy (NST) with trastuzumab in combination with an anthracycline- or a non-anthracycline-based regimen. The pCR rates were 60.6 and 43.3% for patients who received PH-FECH (n = 235) and TCH (n = 65), respectively (*P* = 0.016). The three-year RFS rates were 93 and 71% (*P* < 0.001), and the 3-years OS rates were 96% and 86% (P = 0.008) for patients who received PH-FECH and TCH, respectively. Patients who received PH-FECH had a lower risk of recurrence (hazard ratio [HR]: 0.27; 95% CI: 0.12–0.60; *P* = 0.001) and death (HR: 0.37; 95% CI: 0.12–1.13; *P* = 0.08) than those treated with TCH. Chen et al. [20] observed the efficacy of neoadjuvant trastuzumab combined with docetaxel and carboplatin (TCH), and docetaxel, epirubicin and cyclophosphamide (TEC) chemotherapy in human epidermal growth factor receptor-2 (HER-2)-overexpressing breast cancer. The TCH group comprised 39 patients, and the TEC group comprised 25 patients. Neoadjuvant chemotherapy was continued for six cycles prior to comparison of the treatment efficacy.

### Study strengths and limitations

1. In this study, it was assumed that the transfer probability of the Markov model did not change during the study period. However, in the actual treatment process, the transfer probability between different states changed with time.
2. The incidence of adverse reactions in different disease states of breast cancer was different, and the incidence of adverse reactions generally changed with time. The clinical truth data relied on by this study only provided the incidence of adverse reactions in the entire treatment group without providing the incidence of adverse reactions in different disease states.
3. Due to the lack of research results on the health utility of different states of breast cancer in China, this study adopted the results of Ward et al.[13]

## Conclusion

According to the traditional economic evaluation method, the cost of TCH chemotherapy is lower than that of the AC-TH regimen. Regarding the effect, the clinical yield of the TCH plan was higher than that of the AC-TH plan, and the median survival time of TCH plan was longer than that of the AC-TH plan. Considering the cost and effect, the TCH plan is the best choice in both short-term and long-term economic evaluation. Incremental cost effect analysis showed that the TCH scheme is the preferred scheme with AC-TH as the reference group.

## Data Availability

Not applicable.
